# Ellagic Acid Prevents L-NAME-Induced Hypertension via Restoration of eNOS and p47^phox^ Expression in Rats

**DOI:** 10.3390/nu7075222

**Published:** 2015-06-30

**Authors:** Thewarid Berkban, Pattanapong Boonprom, Sarawoot Bunbupha, Jariya Umka Welbat, Upa Kukongviriyapan, Veerapol Kukongviriyapan, Poungrat Pakdeechote, Parichat Prachaney

**Affiliations:** 1 Department of Anatomy, Faculty of Medicine, Khon Kaen University, 40002 Khon Kaen, Thailand; E-Mails: no_ng_pt@hotmail.com (T.B.); ton_pt_kku@hotmail.com (P.B.); jariya@kku.ac.th (J.U.W.); 2 Department of Physiology, Faculty of Medicine, Khon Kaen University, 40002 Khon Kaen, Thailand; E-Mails: bugvo@hotmail.com (S.B.); upa_ku@kku.ac.th (U.K.); ppoung@kku.ac.th (P.P.); 3 Department of Pharmacology, Faculty of Medicine, Khon Kaen University, 40002 Khon Kaen, Thailand; E-Mail: veerapol@kku.ac.th

**Keywords:** ellagic acid, high blood pressure, nitric oxide deficiency, NADPH oxidase, antioxidant

## Abstract

The effect of ellagic acid on oxidative stress and hypertension induced by N^ω^-Nitro-l-arginine methyl ester hydrochloride (L-NAME) was investigated. Male Sprague-Dawley rats were administrated with L-NAME (40 mg/kg/day) for five weeks. L-NAME induced high systolic blood pressure (SBP) and increased heart rate (HR), hindlimb vascular resistance (HVR) and oxidative stress. Concurrent treatment with ellagic acid (7.5 or 15 mg/kg) prevented these alterations. Co-treatment with ellagic acid was associated with up-regulation of endothelial nitric oxide synthase (eNOS) protein production and alleviation of oxidative stress as indicated by decreased superoxide production in the vascular tissue, reduced plasma malondialdehyde levels, reduced NADPH oxidase subunit p47^phox^ expression and increased plasma nitrate/nitrite levels. Our results indicate that ellagic acid attenuates hypertension by reducing NADPH oxidase subunit p47^phox^ expression, which prevents oxidative stress and restores NO bioavailability.

## 1. Introduction

Hypertension is one of the most important factors associated with development of several diseases such as heart failure, renal failure [1], coronary heart disease, atherosclerosis, myocardial infarction [2] and stroke [3]. Endothelial dysfunction, which results from nitric oxide (NO) deficiency, is one of the causes of essential hypertension [4]. NO, derived from endothelial cells in response to physiological and pathological stimuli, mediates vasodilation, maintains vascular tone and regulates blood pressure [5,6]. Other physiological functions of NO have also been reported such as anti-inflammatory, anti-platelet aggregation, and anti-proliferative effects [7,8,9]. Thus, loss or decrease of NO production plays an important role in pathogenesis of diseases including hypertension, atherosclerosis, myocardial fibrosis, myocardial infarction, diabetes and stroke [10,11,12].

A rat model of hypertension induced by N^ω^-Nitro-l-arginine methyl ester hydrochloride (L-NAME), a nitric oxide synthase (NOS) inhibitor, is widely used to mimic hypertension in humans [13,14]. L-NAME-treated rats have down-regulated eNOS protein expression in blood vessels [15,16] and depletion of plasma NO levels, which leads to systemic vasoconstriction, increased vascular resistance and high blood pressure. There is also an association between L-NAME-induced hypertension and oxidative stress markers. High-dose L-NAME treatment (40 mg/kg/day) has been reported to elevate levels of oxidative stress markers such as vascular superoxide (O_2_^•−^), plasma malondialdehyde (MDA) and plasma protein carbonyl [17,18]. It has been well documented that oxidative stress contributes to the etiology of hypertension in animals [19] and humans [20,21] as characterized by the increased bioavailability of reactive oxygen species (ROS) in hypertension [22]. Elevated ROS levels stimulate vascular smooth muscle cell proliferation and increase arterial resistance due to reduced NO availability, leading to the impairment of vascular relaxation [10]. There is evidence that it is the ROS-producing enzyme, β-Nicotinamide adenine dinucleotide phosphate (NADPH) oxidase (NOx), that is responsible for increased vascular O_2_^•−^ production in L-NAME hypertensive rats [23] via up-regulation of the NOx subunit p47^phox^ [24].

Nowadays, there is a great deal of interest in how antioxidant substances can be used for prevention and treatment of disease. Ellagic acid is a natural polyphenolic compound present in oak species such as the North American white oak (*Quercus alba*) and European red oak (*Quercus robur*) [25]. Ellagic acid is also found in fruits, such as blackberries, cranberries, raspberries, strawberries, grapes, pomegranates, and nuts, as well as several medicinal plants [26,27,28]. Previous studies have revealed that ellagic acid possesses anticancer [27,29], antioxidant [30,31,32] and anti-inflammatory activities [33] and can aid in the prevention of degenerative diseases such as cardiovascular disease [34]. However, little is known regarding the antihypertensive effects of ellagic acid. This study aims to examine whether ellagic acid can reduce blood pressure and oxidative stress markers in L-NAME-induced hypertensive rats.

## 2. Materials and Methods

### 2.1. Reagents

Ellagic acid (purity ≥ 95%), L-NAME, ethylenediaminetetraacetic acid (EDTA), butylated hydroxytoluene (BHT), thiobarbituric acid (TBA), sodium dodecyl sulfate (SDS), sulfanilamide, N-1-napthylethylethylenediamine (NED) and 1,1,3,3-tetraethoxypropane were obtained from Sigma-Aldrich Corp. (St. Louis, MO, USA). β-Nicotinamide adenine dinucleotide phosphate (NADPH), glucose-6-phosphate dehydrogenase (G-6-PD) and Nitrate reductase were obtained from Roche Applied Sciences (Mannheim, Germany). Trichloroacetic acid (TCA) and lucigenin were obtained from Fluka Chemika Co., Ltd (Buch, Switzerland). All chemicals used in this study were obtained from standard companies and were of analytical grade quality.

### 2.2. Instruments

Pressure transducer equipped with AcqKnowledge data acquisition and analysis software (BIOPAC Systems Inc., Santa Barbara, CA, USA), Electromagnetic flow meter (Model FM501D, Carolina Medical Electronics, Carolina, NC, USA), tail-cuff plethysmography (IITC/Life Science Instrument model 229 and model 179 amplifiers, Woodland Hills, CA, USA), microplate reader (Tecan GmbH., Groding, Austria), UV/Visible spectrophotometer (Ultrospec 6300 Pro. Bichrom Ltd. UK), luminometer (Turner Biosystems, 23 CA, USA), Centrifuge SIGMA 3K15 (SIGMA Laborzentrifugen, Osterode am Harz, Germany) and Centrifugal concentrators (NANOSEP™, Pl Filtration, USA) were used in this study.

### 2.3. Animals and Experimental Protocols

#### 2.3.1. Animals

Male Sprague-Dawley rats (240–280 g) were obtained from the National Laboratory Animal Center, Mahidol University, Salaya, Nakornpathom. The rats were housed at 25.1 ± 1 °C with 12 h dark–light cycle at Northeast Laboratory Animal Center, Khon Kaen. All procedures were reviewed and approved by the Institutional Animal Ethics Committee of Khon Kaen University (AEKKU 70/2555).

#### 2.3.2. Induction of L-NAME Hypertension

The rats in the L-NAME-treated group were fed with standard chow diet (Chareon Pokapan Co., Bangrak, Thailand) and L-NAME (40 mg/kg/day) in their drinking water for 5 weeks to induce hypertension, whereas rats in the normal control group were fed with a standard chow diet and distilled water (DW).

#### 2.3.3. Experimental Groups

After seven days of acclimatization, the rats were randomly divided into five experimental groups (*n* = 10/group) as follows.

Group 1: Control (DW)
Group 2: Control + EA 15 (Ellagic acid 15 mg/kg BW in DW)
Group 3: L-NAME (DW)
Group 4: L-NAME + EA 7.5 (Ellagic acid 7.5 mg/kg BW in DW)
Group 5: L-NAME + EA 15 (Ellagic acid 15 mg/kg BW in DW)

Ellagic acid and/or distill water vehicle were intragastrically administered daily. The doses of ellagic acid were chosen on the basis of previous studies [35]. Blood pressure was measured before entering the study and during treatment until sacrifice. Body weight was measured weekly and the dose was adjusted accordingly.

### 2.4. Parameter Measurements

#### 2.4.1. Blood Pressure Measurement

Systolic blood pressure (SBP) was measured once a week using non-invasive tail-cuff plethysmography (IITC/Life Science Instrument model 229 and model 179 amplifiers, Woodland Hills, CA, USA). Briefly, conscious rats were placed in a restrainer and allowed to rest for 10–15 min prior to blood pressure measurement. The tail was placed inside the cuff, which automatically inflated and released and SBP values were obtained from the mean of three measurements.

#### 2.4.2. Hemodynamic Assessments

On the last day of the experiment, rats were anaesthetized with an intraperitoneal injection of Pentobarbital (60 mg/kg) and placed on heating pad to maintain body temperature at 37 °C. A tracheotomy was performed for measurement of spontaneous breathing, and a polyethylene catheter was inserted into the lower abdominal aorta via the left femoral artery for continuous monitoring of blood pressure using a pressure transducer and the Acknowledge data acquisition and analysis software (BIOPAC Systems Inc., Santa Barbara, CA, USA). The catheters were filled with heparinized saline to prevent clotting. SBP, diastolic blood pressure (DBP), mean arterial blood pressure (MAP) and heart rate (HR) were continuously monitored.

Hindlimb blood flow (HBF) was continuously measured by an electromagnetic flow meter (Carolina Medical Electronics, Carolina, NC, USA) connected to an electromagnetic flow probe placed around the abdominal aorta below the kidneys. Hindlimb vascular resistance (HVR) was calculated from baseline MAP and mean HBF.

After collection of the hemodynamic measurements, rats were sacrificed with an over dosage of the anesthetic drug. Blood samples were drawn from the bifurcation of the abdominal aorta into EDTA tubes for assay of the oxidative stress markers plasma MDA and NO metabolites. The carotid arteries were rapidly excised for analysis of vascular O_2_^•−^ production and the thoracic aorta was isolated to evaluate eNOS and NADPH oxidase subunit (p47^phox^) protein expression.

#### 2.4.3. Assay of Vascular O_2_^•−^ Production

Vascular O_2_^•−^ production was measured using a lucigenin-enhanced chemiluminescence method [36]. The carotid arteries (about 3–5 mm in length) were placed in ice-cold saline and adipose and connective tissues were removed. The vessel segments were incubated with Krebs-KCL buffer (pH 7.4) and allowed to equilibrate at 37 °C for 30 min. Lucigenin (100 μM) was added and the sample tube placed in a luminometer (Turner Biosystems, Sunnyvale, CA, USA). The photon counts were integrated every 30 s for 5 min and averaged. The vessels were then dried at 45 °C for 24 h for determination of dry weight. O_2_^•−^ production in vascular tissue was expressed as relative light unit count per minute per milligram of dry tissue weight.

#### 2.4.4. Assay of Plasma Malondialdehyde

The concentration of plasma MDA was measured by a spectrophotometric method as previously described [37]. Briefly, plasma samples (150 µL) were reacted with 10% TCA, 125 μL of 5 mM EDTA, 125 μL of 8% SDS, and 10 μL of 0.5 µg/mL BHT for 10 minutes at room temperature. Then, an equal volume of 0.6% TBA was added and the mixture was boiled in a water bath for 30 min. After cooling to room temperature, the mixture was centrifuged at 10,000 g for 5 min at 25 °C. The absorbance of the supernatant was measured at 532 nm by spectrophotometer (Amersham Bioscience, Arlington, MA, USA) and compared to a standard curve generated with 0.3 to 10 µmol/L 1,1,3,3-tetraethoxypropane.

#### 2.4.5. Assay of Plasma Nitrate and Nitrite

The concentrations of plasma nitrate and nitrite, the end products of NO metabolism, were measured using an enzymatic conversion method [38] with some modifications [39]. Plasma samples were first deproteinized by ultrafiltration using centrifugal concentrators (Pall Corp., Ann Arbor, MI, USA). The supernatants were mixed with 1.2 µmol/L NADPH, 4 mmol/L glucose-6-phosphate disodium (G-6-P), 1.28 U/mL glucose-6-phosphate dehydrogenase (G-6-PD) and 0.2 U/mL nitrate reductase, and then incubated at 30 °C for 30 min. The mixture was reacted with a Griess solution (4% sulfanilamide in 0.3% naphthalenediamine dichloride, NED) for 15 min. The absorbance of samples was measured on a microplate reader with a filter wavelength of 540 nm (Tecan GmbH., Groding, Austria). 

#### 2.4.6. Western Blot Analysis of p47^phox^ NADPH Oxidase Subunit and eNOS Protein

Expressions of the p47^phox^ NADPH oxidase subunit and eNOS proteins in aortic homogenates were determined following a previously described method with some modifications [17,40]. The aortic homogenates (15 µg) were electrophoresed on an SDS polyacrylamide gel and electrotransferred onto a polyvinylidenedifluoride membrane. Membranes were blocked with 5% skimmed milk in phosphate buffered saline (PBS) with 0.1% Tween-20 at room temperature for 2 h and incubated overnight at 4 °C with primary antibody of mouse monoclonal anti-eNOS (1:2500, BD Biosciences, CA, USA) or mouse monoclonal anti-p47^phox^ (1:2500, Santa Cruz Biotechnology, CA, USA). The membranes were washed with PBS 3 times for 7 min before being incubated with the secondary antibody, horseradish peroxidase goat anti-mouse IgG (1:2500, Santa Cruz Biotechnology, CA, USA), for 2 h at room temperature. The blots were developed in Amersham™ ECL™ Prime solution (Amersham Biosciences Corp., Piscataway, NJ, USA), and densitometric analysis was performed using an ImageQuant™ 400 imager (GE Healthcare Life Sciences, Piscataway, NJ, USA). β-actin (1:5000, Santa Cruz Biotechnology, CA, USA) was used as a loading control. The intensity of the specific eNOS and p47^phox^ protein bands were normalized to that of β-actin, and data were expressed as a percentage of the values determined in control aorta from the same gel.

### 2.5. Statistical Analysis

Data were expressed as mean ± SEM. The significance of differences between means were analyzed by one-way analysis of variance (ANOVA) and followed by Student Newman–Keul’s test to show specific group differences. Statistical significance was assigned at a *p* value of less than 0.05.

## 3. Results

### 3.1. Effect of Ellagic Acid on Systolic Blood Pressure

**Figure 1:**
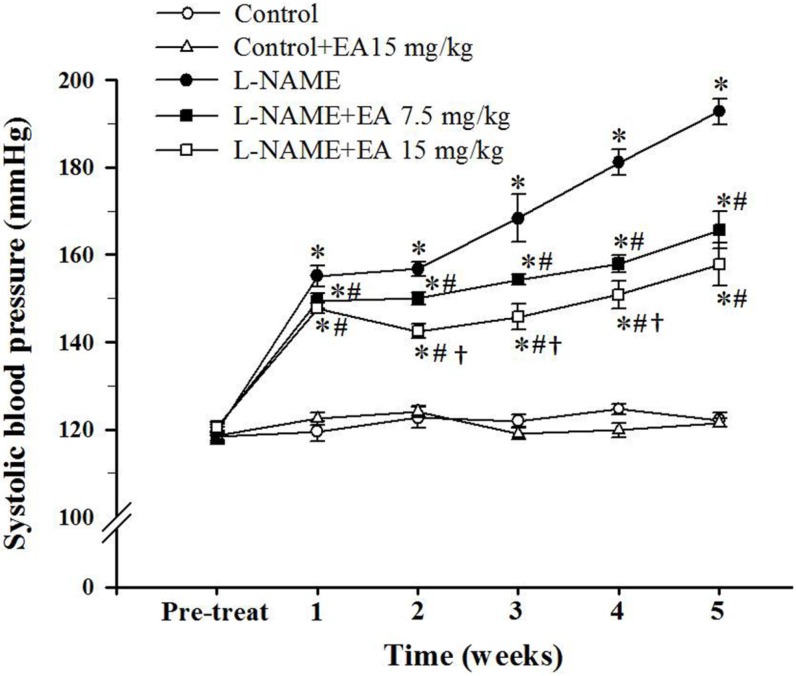
Effect of ellagic acid on systolic blood pressure during N^ω^-Nitro-l-arginine methyl ester hydrochloride (L-NAME) administration for five weeks. Values are expressed as mean ± SEM, *n* = 8. * *p* < 0.05 when compared to control group, ^#^ *p* < 0.05 when compared to L-NAME group and ^†^ *p* < 0.05 when compared to L-NAME+EA 7.5 mg/kg group.

At the beginning of the experiment, average baseline SBP was similar among groups (Figure 1). L-NAME administration induced a rapid progressive increase in SBP. Rats receiving L-NAME with concurrent administration of ellagic acid showed increased SBP compared to controls but reduced SBP compared to L-NAME treatment alone. The L-NAME+EA 15 mg/kg/day group showed a significantly lower SBP than the L-NAME+EA 7.5 mg/kg/day group (157.8 ± 4.8 mmHg *vs.* 165.7 ± 4.3, *p* < 0.05).

### 3.2. Effect of Ellagic Acid on Hemodynamic Status

Animals that received only L-NAME were hypertensive as evidenced by significant increases in SBP, DBP, MAP and HR compared to control rats (Table 1). Concurrent treatment with ellagic acid significantly attenuated the elevation of the BP parameters and HR in L-NAME hypertensive rats (Table 1). L-NAME administered rats also showed significantly lower HBF levels and significantly higher HVR levels compared with control rats (4.2 ± 0.3 *vs.* 8.3 ± 0.8, *p* < 0.05 and 39.5 ± 3.5 *vs.* 12.1 ± 1.4, *p* < 0.001, respectively). Both the 7.5 mg/kg and 15 mg/kg doses of ellagic acid ameliorated the L-NAME induced changes in HBF and HVR in hypertensive rats (Table 1).

**Table 1:** Effect of ellagic acid on cardiovascular parameters and body weight.

Parameters	Control			l-NAME	
	Vehicle	EA (mg/kg/day)	Vehicle		EA (mg/kg/day)
		15		7.5	15
SBP (mmHg)	121.9 ± 1.8	122.7 ± 2.3	199.4 ± 6.1 *	167.5 ± 3.0 *^,#^	164.6 ± 4.9 *^,#^
DBP (mmHg)	78.3 ± 2.0	78.8 ± 2.1	140.4 ± 4.0 *	113.6 ± 3.1 *^,#^	111.2 ± 3.7 *^,#^
MAP (mmHg)	95.4 ± 1.9	95.5 ± 2.2	164.3 ± 4.6 *	136.8 ± 3.2 *^,#^	133.1 ± 4.2 *^,#^
HR (beat/min)	357.9 ± 6.0	362.5 ± 5.9	424.8 ± 6.2 *	379.4 ± 5.5 *^,#^	368.0 ± 3.1 *^,#^
HBF (mL/100 g tissue/min)	8.3 ± 0.8	7.1 ± 0.4	4.2 ± 0.3 *	6.4 ± 0.2 *^,#^	6.4 ± 0.3 *^,#^
HVR (mmHg/min/100 g/mL)	12.1 ± 1.4	13.8 ± 0.8	39.5 ± 3.5 *	20.5 ± 1.2 *^,#^	20.4 ± 1.2 *^,#^
Body weight (g)	417.1 ± 4.6	418.0 ± 6.0	409.5 ± 2.6	414.4 ± 4.4	410.5 ± 4.3

Values are expressed as mean ± SEM, *n* = 10. * *p* < 0.05 when compared to control group and ^#^ *p* < 0.05 when compared to vehicle L-NAME group.

### 3.3. Effects of Ellagic on Nitrate/Nitrite Production and eNOS Protein Expression

**Figure 2:**
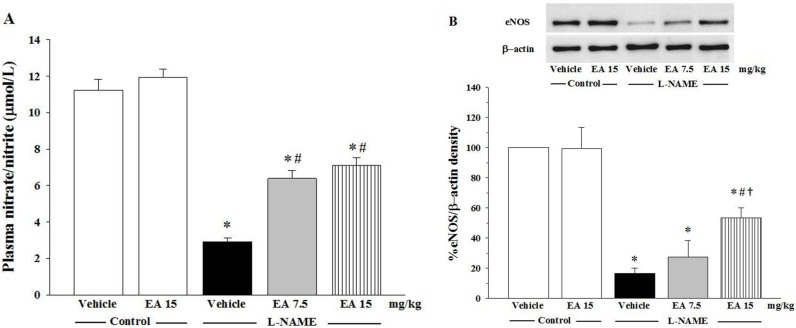
Effect of ellagic acid on (**A**) nitrate/nitrite production (*n* = 7) and (**B**) eNOS protein expression (*n* = 5). Values are expressed as mean ± SEM, * *p* < 0.05 when compared to control group, ^#^ *p* < 0.05 when compared to L-NAME group and ^†^ *p* < 0.05 when compared to L-NAME+EA 7.5 mg/kg group.

The concentration of nitrate/nitrite NO metabolites in plasma is shown in Figure 2A and the expression of eNOS protein in isolated rat aortas is shown in Figure 2B. The levels of NO metabolites in plasma were significantly reduced in L-NAME rats when compared with normal control rats (2.9 ± 0.2 µmol/L *vs.*11.2 ± 0.6 µmol/L, *p* < 0.05) and administration of L-NAME was associated with down-regulation of eNOS protein expression compared to control rats (*p* < 0.05). There was no change in plasma nitrate/nitrite levels or eNOS protein expression in control rats treated with ellagic acid alone (Figure 2A,B). Co-treatment of L-NAME administered rats with either 7.5 mg/kg or 15 mg/kg ellagic acid for five weeks significantly restored nitrate/nitrite production (6.4 ± 0.5 µmol/L *vs.* 7.1 ± 0.4 µmol/L, *p* < 0.05, respectively) and eNOS protein expression, *p* < 0.05, respectively).

### 3.4. Effect of Ellagic Acid on Oxidative Stress

**Figure 3:**
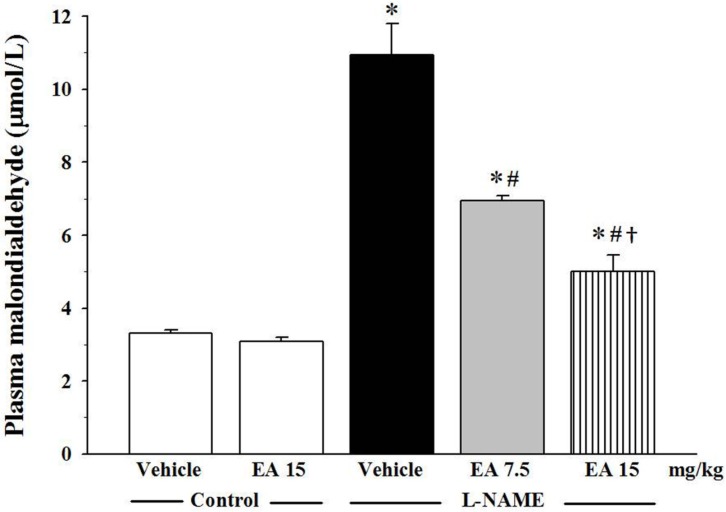
Effect of ellagic acid on plasma malondialdehyde levels. Values are expressed as mean ± SEM, *n* = 7. * *p* < 0.05 when compared to control group, ^#^ *p* < 0.05 when compared to L-NAME group and ^†^ *p* < 0.05 when compared to L-NAME+EA 7.5 mg/kg group.

**Figure 4:**
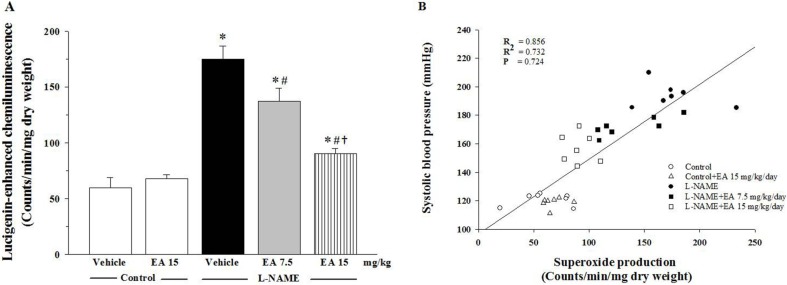
(**A**) Effect of ellagic acid on vascular superoxide production. Values are expressed as mean ± SEM lucigenin-enhanced chemilumiescence counts/min/mg dry weight, *n* = 7. * *p* < 0.05 when compared to control group, ^#^ *p* < 0.05 when compared to L-NAME group and ^†^ *p* < 0.05 when compared to L-NAME+EA 7.5 mg/kg group. (**B**) Correlation between SBP and vascular superoxide production.

Oxidative stress was determined by measuring the concentration of plasma MDA and by assaying vascular superoxide production. The level of plasma MDA in the L-NAME group was three-fold higher than in the control group (10.9 ± 0.9 µmol/L *vs.* 3.3 ± 0.1 µmol/L, *p* < 0.05). Administration of both 7.5 and 15 mg/kg ellagic acid significantly reduced the level of plasma MDA in a dose dependent manner (6.9 ± 0.1 µmol/L and 5.0 ± 0.5 µmol/L, *p* < 0.05, respectively, Figure 3). L-NAME also induced a three-fold elevation in vascular superoxide production compared to the vehicle control (175.3 ± 11.2 count/min/mg dry weight *vs.* 59.9 ± 8.9 count/min/mg dry weight, *p* < 0.05) (Figure 4A). Again, ellagic acid at both the 7.5 and 15 mg/kg doses significantly inhibited this elevation (137.1 ± 11.8 count/min/mg dry weight and 90.4 ± 4.6 count/min/mg dry weight, *p* < 0.05, respectively). In addition, there was a strong correlation between vascular superoxide production and SBP (*R* = 0.856; Figure 4B).

### 3.5. Effect of Ellagic Acid on p47^phox^ Protein Expression in Aortic Tissues

**Figure 5:**
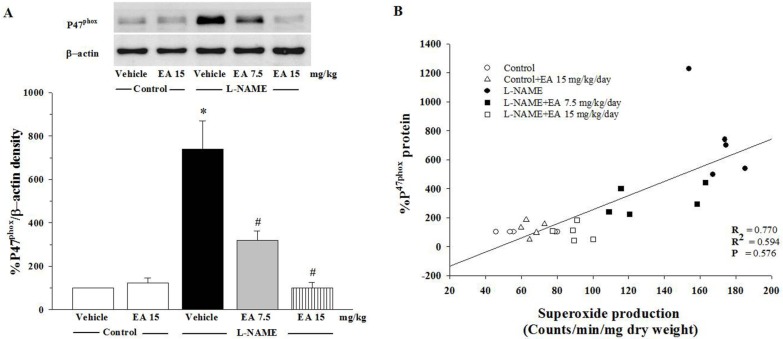
(**A**) Effect of EA on p47^phox^ protein expression in aortic tissues. Values are expressed as mean ± SEM, *n* = 5. * *p* < 0.05 when compared to control group and ^#^ *p* < 0.05 when compared to L-NAME group. (**B**) Correlation between p47^phox^ protein expression and vascular superoxide production.

The expression of p47^phox^ protein in L-NAME treated rats was significantly up-regulated compared to control rats (*p* < 0.05, Figure 5A). Positive correlation of p47^phox^ protein expression and the values of the vascular O_2_^•−^ production was observed (Figure 5B). Treatment of the L-NAME group with 7.5 mg/kg ellagic acid reduced the over-expression of p47^phox^ protein (*p* < 0.05) and treatment with 15 mg/kg ellagic acid restored p47^phox^ protein levels to normal (Figure 5A).

## 4. Discussion

The present study examined the antihypertensive effects of ellagic acid and possible mechanisms involved in L-NAME-induced hypertensive rats. The findings demonstrate that L-NAME-induced hypertension exhibited hemodynamic alterations, including high blood pressure, high HVR, and low HBF. There was a reduction of plasma NOx that was consistent with down-regulation of eNOS expression in hypertensive rats. Oxidative stress markers were significantly increased in this animal model of experimental hypertension. The protein expression of p47^phox^ NADPH oxidase subunit was markedly increased in hypertensive rats. Administration of ellagic acid reduced blood pressure, HR, HVR as well as increased HBF in L-NAME hypertensive rats. Decreased plasma NOx and eNOS expression in hypertensive rats were normalized by daily treatment with ellagic acid. Furthermore, ellagic acid also reduced plasma MDA, vascular O_2_^•−^ production and alleviated the expression of the NADPH oxidase subunit in these rats. 

It is well established that long-term administration of the L-arginine analogue L-NAME to normotensive rats can induce NO-deficient hypertension. The precise mechanism is based on the fact that NO is synthesized and released from endothelial cells to mediate vasorelaxation [6] and L-NAME reduces NO production resulting in increased total peripheral resistance and high blood pressure [41,42]. This mechanism is supported by findings that disruption of plasma NOx level and reduced protein eNOS expression have been reported in long-term L-NAME treatment [15,43,44].

Our results demonstrate that L-NAME treatment for five weeks caused a progressive increase in blood pressure resulting from increased HR and HVR. These observations were also associated with a reduction in plasma NOx levels and down-regulation of protein eNOS expression. The doses of ellagic acid (7.5 and 15 mg/kg/day) chosen for this study represent pharmacological doses. The higher dose of 15 mg/kg/day would equate to human consumption of approximately 850 mL/day of pomegranate juice [45,46]. However, this dose did not lead to any evident toxicity as indicated by the fact that the body weight of these rats was not different to that of the vehicle-treated rats. Moreover, our results showed that ellagic acid markedly improved the symptoms of hypertension in L-NAME treated rats. The protective effect of ellagic acid was characterized by reductions in HR and HVR and augmentation of HBF. While this report is the first to directly demonstrate the protective effect of ellagic acid on L-NAME-induced hypertension, several previous studies have shown beneficial effects of consumption of pomegranate juice, which contains a high concentration of ellagic acid [31,47,48]. For example, Aviram and coworkers found that consumption of pomegranate juice (50 mL/day) for a year was able to reduce SBP in atherosclerotic patients and they suggested that the pomegranate juice had a potent antioxidative capacity as one possible mechanism [47]. In another study, NOS activity was improved in diet-induced atherosclerosis in mice after ellagic acid supplementation [48]. Finally, in diabetic Wistar rats, chronic administration of pomegranate juice extract (100 and 300 mg/kg/day) for four weeks caused a reduction in MAP presumably related to its antioxidant capacity [31].

Oxidative stress is an imbalance between the production of reactive oxygen species and the antioxidant capacity, and has been found to link with the pathogenesis of hypertension [19,20,21]. The association between oxidative stress and hypertension has been extensively demonstrated ever since the plasma level of MDA, a biomarker of lipid peroxidation, was found to be increased in patients with hypertension [49,50,51]. In L-NAME hypertensive rats, we found increases in the level of plasma MDA and vascular O_2_^•−^. In addition, a positive correlation between SBP and vascular O_2_^•−^ production was shown in this study, suggesting that O_2_^•−^ rapidly reacts with NO to produce peroxynitrite (ONOO^−^) resulting in impaired NO bioavailability [52]. These findings are supported by several studies in L-NAME hypertensive rats [44,53,54]. The increase in O_2_^•−^ production in L-NAME hypertensive rats is probably due to the augmentation of NADPH oxidase activity, a major source of vascular O_2_^•−^ production. It has been well documented that O_2_^•−^ production is primarily produced from NADPH oxidase, which has several subunits [23]. In this study, we found the up-regulation of p47^phox^ protein expression, an NADPH oxidase subunit, in the vascular tissues. This observation has been reported that in nitric oxide deficient hypertensive rats showed an over expression of NADPH oxidase subunit p47^phox^ with lipid peroxidation and high levels of vascular O_2_^•−^ production [24,55]. Thus, our findings suggested that increased vascular O_2_^•−^ level observed in L-NAME hypertensive rats play an important role for decreasing NO bioavailability as it was confirmed by the low level of plasma NOx. 

Here we report that ellagic acid reduced the development of L-NAME-induced hypertension in rats by significantly reducing oxidative stress markers, and NADPH oxidase subunit p47^phox^ expression. There is substantial evidence to show that ellagic acid has potent antioxidative effects. Goswami and coworkers found that dietary supplementation with reduced oxidative stress and improved sexual dysfunction in type 1 diabetic rats [56]. In addition, ellagic acid was reported to prevent diet-induced atherosclerosis in WT mice and this was related to improvements in NO availability, reduced oxidative stress and activation of the Nrf2 antioxidant transcription factor [48]. It has also been reported that oxidation can alter the extracellular matrix of the arterial wall [57] and changes to the arterial wall have been implicated as a key factor in the pathogenesis of hypertension [58]. Therefore, any effects of ellagic acid on the extracellular matrix of the vascular wall of the L-NAME induced-hypertensive rat should be investigated.

## 5. Conclusions

This study demonstrates that ellagic acid exhibits protective effects on the development of hypertension induced by a NOS inhibitor. These protective effects are associated with improved NO bioavailability, higher eNOS protein expression and reduced oxidative stress status, possibly due to a reduction in NADPH oxidase subunit p47^phox^ expression.
